# Supplementary material for: Non-invasive monitoring of alternative splicing outcomes to identify candidate therapies for myotonic dystrophy type 1
Source: Nat Commun. 2018 Dec 7;9:5227. doi: 10.1038/s41467-018-07517-y (PMC6286378; doi:10.1038/s41467-018-07517-y)
Supplement: Supplementary file 1 — Supplementary Information [file 41467_2018_7517_MOESM1_ESM.pdf]

Non-invasive monitoring of alternative splicing outcomes to identify candidate therapies for myotonic dystrophy type 1

Ningyan Hu[1,2], Layal Antoury[1,2], Timothy M. Baran[3], Soumya Mitra[3], C. Frank Bennett[4], Frank Rigo[4], Thomas H. Foster[3], and Thurman M. Wheeler[1,2,*]

[1]Department of Neurology, Massachusetts General Hospital, Boston, MA, USA. [2]Harvard Medical School, Boston, MA, USA. [3]Department of Imaging Sciences, University of Rochester, Rochester, NY, USA. [4]Ionis Pharmaceuticals, Carlsbad, CA, USA.

[*]Corresponding author:

twheeler1@mgh.harvard.edu

Contents:

Supplementary Figures 1 - 13

Supplementary Tables 1 - 4

Supplementary Note 1

Supplementary References

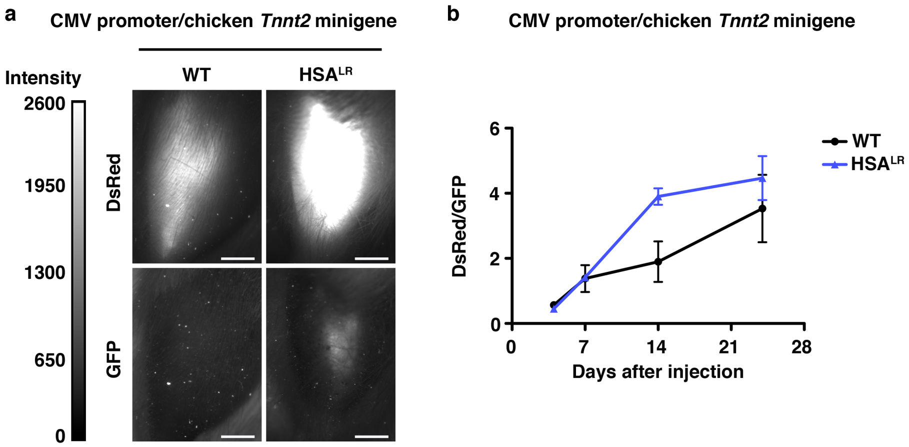


**Supplementary Figure 1** Expression of bi-chromatic reporter plasmid DNA in skeletal muscle. A previously published bi-chromatic reporter construct determines expression of either DsRed or GFP by splicing of an upstream chicken cardiac troponin T (*Tnnt2*) minigene[1]. We injected and electroporated[2] 20 μg plasmid DNA containing this construct into tibialis anterior (TA) muscles of FVB wild-type (WT) and the human skeletal actin-long repeat ($HSA^{LR}$) transgenic mouse model of DM1 (N = 3 each), and monitored fluorescence protein expression by non-invasive *in vivo* fluorescence microscopy of live mice. (**a**) Representative DsRed (upper) and GFP (lower) images of TA muscles in WT and $HSA^{LR}$ mice on Day 14 after plasmid injection. Fluorescence intensity range = 0 - 2600 grayscale units. Bars = 2 millimeters. (**b**) Quantitation of DsRed/GFP ratios by serial *in vivo* fluorescence imaging of WT (black circles) and $HSA^{LR}$ (blue triangles) mice described in (**a**). Error bars indicate mean ± s.e.m.

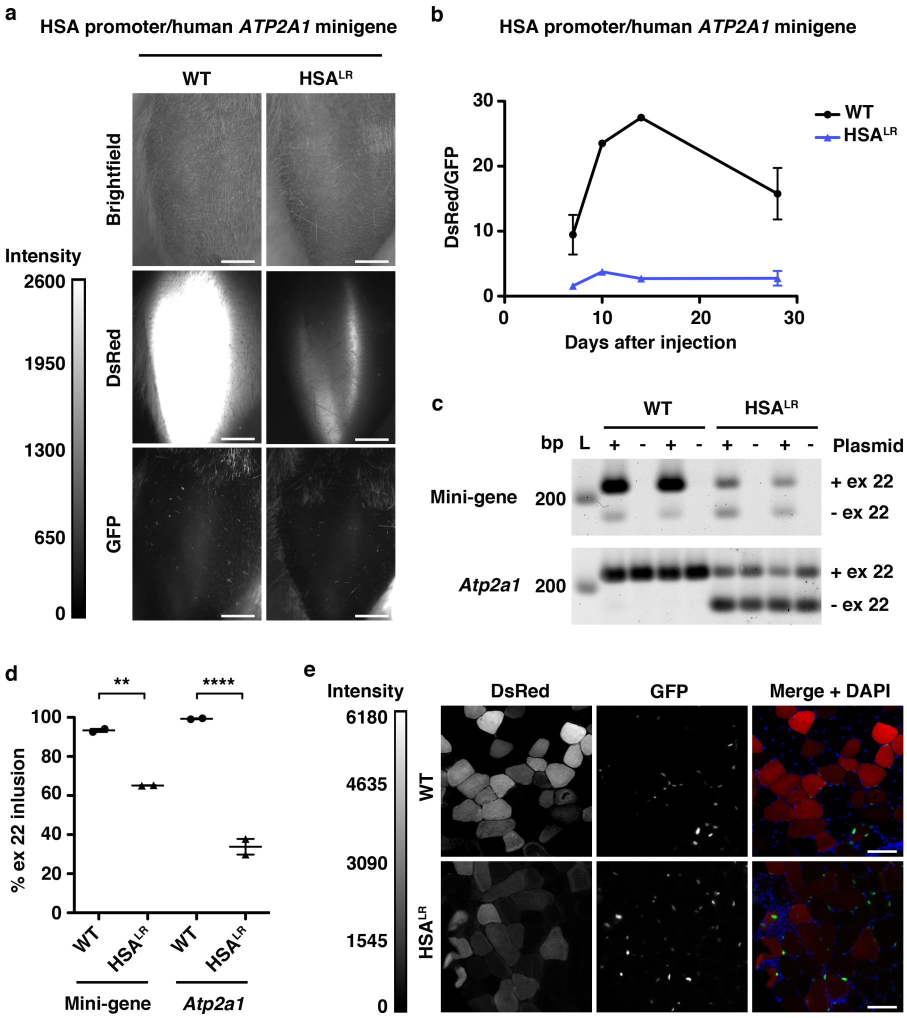


**Supplementary Figure 2** Expression of therapy reporter (TR) plasmid DNA in skeletal muscle. To test the new TR construct (see Fig. 1a), we injected and electroporated 20 μg TR plasmid DNA into TA muscles[2] (N = 2 each), and followed gene expression by serial *in vivo* fluorescence microscopy. The contralateral TA was injected with saline. (**a**) Representative brightfield (upper), DsRed (middle), and GFP (lower) images of TA muscles in WT and *HSA*$^{LR}$ mice on Day 10 after plasmid injection. Fluorescence intensity range = 0 - 2600 grayscale units. Bars = 2 millimeters. (**b**) Quantitative DsRed/GFP ratios in WT (black circles) and *HSA*$^{LR}$ (blue triangles) mice described in (**a**). Error bars indicate mean ± s.e.m. (**c**) RT-PCR analysis of exon 22 alternative splicing of TR plasmid *ATP2A1* minigene (upper) and endogenous mouse *Atp2a1* (lower) at Day 28. (+) = plasmid-treated; (-) = no plasmid (saline-treated). (**d**) Quantitation of splicing results in (**c**). Error bars indicate mean ± s.e.m. **** $P < 0.0001$, ** $P < 0.01$; two-way ANOVA. (**e**) Representative images of quantitative DsRed and GFP fluorescence in TA muscle cryosections 28 days after injection with TR plasmid DNA. DAPI (blue) highlights nuclei. Merge = DsRed (red) + GFP (green) + DAPI. Fluorescence intensity range = 0 - 6180 grayscale units. Bars = 50 μm.

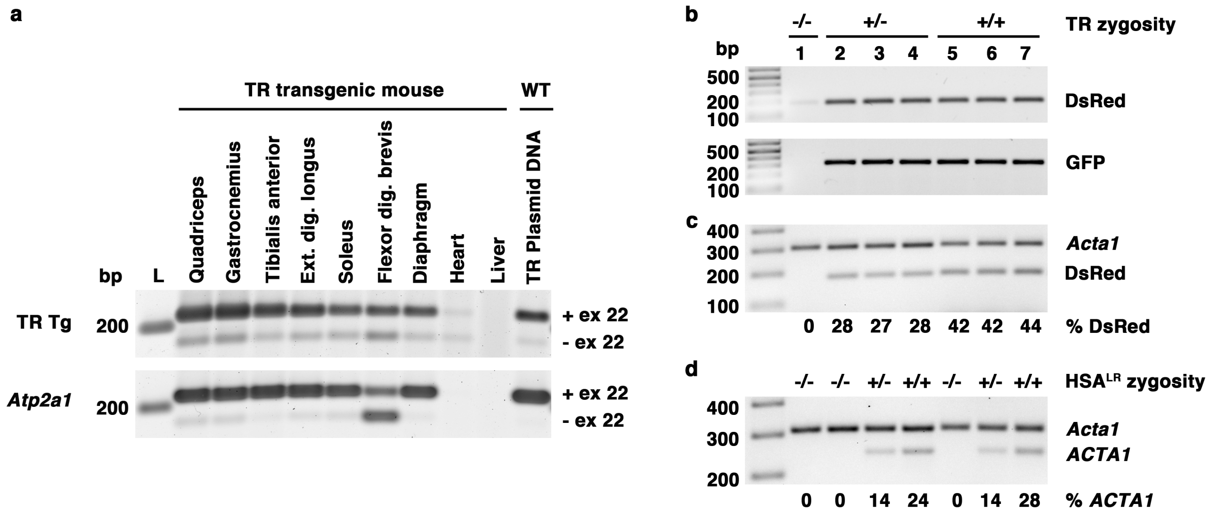


**Supplementary Figure 3** TR transgene splicing pattern, genotyping, and determination of zygosity. (**a**) RT-PCR analysis of the TR transgene and endogenous mouse *Atp2a1* exon 22 splicing pattern in quadriceps, gastrocnemius, tibialis anterior, extensor digitorum longus, soleus, flexor digitorum brevis, and diaphragm muscles, and heart and liver tissues from a TR transgenic mouse. TA muscle of a wild-type (WT) mouse injected with TR plasmid DNA served as a control. Shown are representative results from N = 3 examined. TR transgene expression appears low in the heart and absent in liver, consistent with expression pattern of the HSA promoter/enhancer[3]. (**b**) Genotyping of N = 7 mice by PCR using toe clip tissue (see Methods) and primers targeting the DsRed (upper) or GFP (lower) portion of the transgene. Presence of a band indicates presence of the transgene, but is unable to distinguish hemizygous from homozygous mice. (**c**) To determine zygosity of the TR transgene, we used primers for both DsRed and endogenous mouse *Acta1* in the same PCR reaction. TR hemizygous mice have < 30% DsRed vs. *Acta1*, while TR homozygous mice have > 40 % DsRed. Absence of a DsRed band defines absence of the TR transgene. (**d**) PCR determination of the $HSA^{LR}$ transgene zygosity using primers for the human *ACTA1* transgene and the endogenous mouse *Acta1* gene. $HSA^{LR}$ hemizygous (hemi) mice have < 15 % *ACTA1*, while $HSA^{LR}$ homozygous (hom) mice have > 22% *ACTA1*.

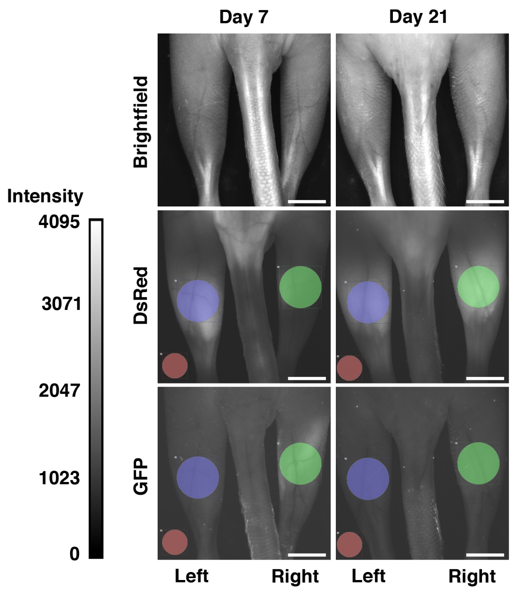


**Supplementary Figure 4** Quantitation of fluorescence by *in vivo* microscopy. To test the splice switch *in vivo*, we injected the right gastrocnemius muscles of homozygous TR transgenic mice with 1.2% barium chloride to induce muscle injury and regeneration (N = 8 total; see Fig. 1). Shown are representative brightfield (upper), DsRed (middle), and GFP (lower) images on Days 7 and 21 after injury. Mice are prone and were imaged under general anesthesia after removal of hair (Nair). The colored circles indicate regions of interest (ROI) where fluorescence intensity was measured in the uninjured left gastrocnemius (blue circles), injured right gastrocnemius (green circles), and background signal (red circles). Fluorescence intensity range = 0 - 4095 grayscale units. Bars = 4 millimeters.

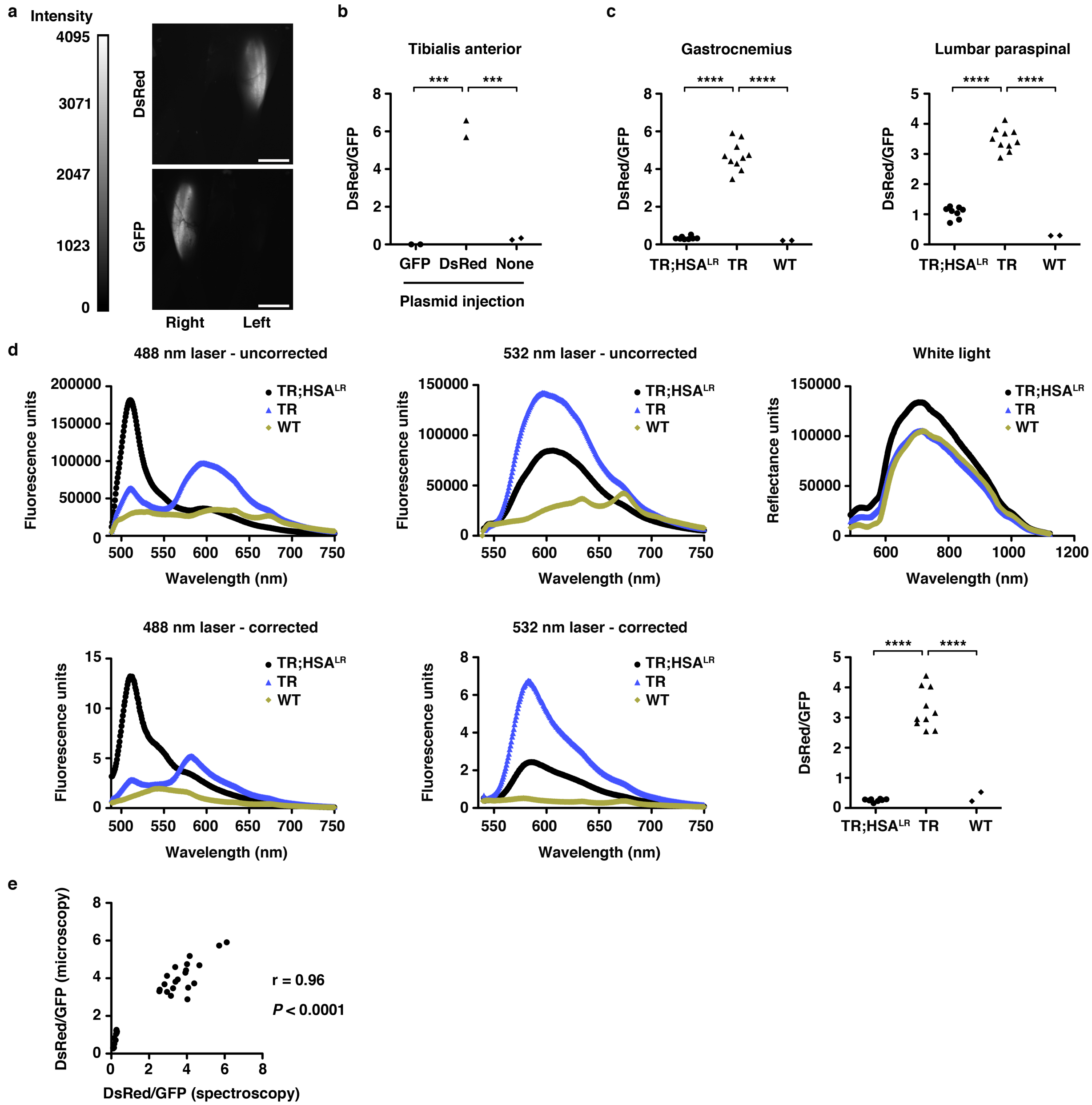


**Supplementary Figure 5** Calibration of *in vivo* fluorescence spectroscopy measurements. (**a**) Representative quantitative fluorescence microscopy of DsRed and GFP 7 days after intramuscular injection of 20 μg DsRed plasmid DNA into the left tibialis anterior (TA) muscle and 20 μg GFP plasmid DNA into the right TA muscle of wild-type (WT) mice (N = 2 each) (see Figure 3c). Fluorescence intensity range = 0 - 4095 grayscale units. Bars = 4 millimeters. (**b**) Quantitative DsRed/GFP values in WT TA muscles injected with DsRed or GFP plasmid DNA (N = 2 each), as measured by *in vivo* microscopy. Uninjected WT TA muscles (N = 2) served as controls. *** $P = 0.0002$; one-way ANOVA. (**c**) Quantitative DsRED/GFP values in gastrocnemius and lumbar paraspinal muscles of TR (N = 5 mice; 10 muscles each), TR;HSA$^{LR}$ (N = 4 mice; 8 muscles each), and WT (N =1 mouse; 2 muscles each), as measured by *in vivo* microscopy. ****$P <$ 0.0001; one-way ANOVA. (**d**) We used spectroscopy to measure *in vivo* fluorescence in lumbar paraspinal

muscles of TR;HSA$^{LR}$ bi-transgenic (black circles) (N = 4 mice; 8 muscles total), TR transgenic (blue triangles) (N = 5 mice; 10 muscles total), and WT control (yellow diamonds) (N = 1 mouse; 2 muscles). Shown are representative uncorrected fluorescence, reflectance, and corrected fluorescence spectra of each, and DsRed/GFP ratios for all muscles examined. *In vivo* fluorescence microscopy data from these muscles are shown in (**c**). **** $P < 0.0001$; one-way ANOVA. (**e**) Correlation of DsRed/GFP values in TR;HSA$^{LR}$, (N = 4 mice, 8 gastrocnemius and 8 paraspinal muscles total), TR (N = 5 mice, 10 gastrocnemius and 10 paraspinal muscles total), and FVB wild-type (N = 1 mouse, 2 gastrocnemius and 2 paraspinal muscles) measured by spectroscopy (x-axis) and microscopy (y-axis) in (**b**), (**c**), and Figure 3. The correlation coefficient r and *P* values are shown.

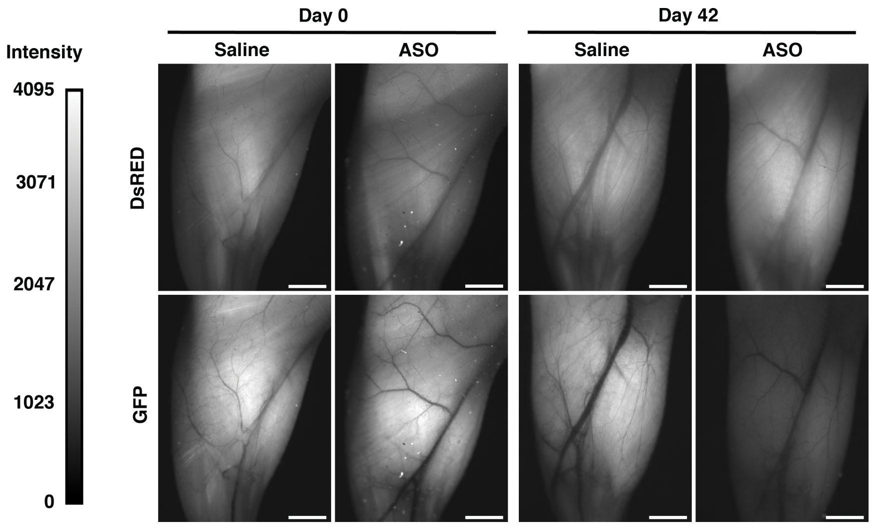


**Supplementary Figure 6** *In vivo* fluorescence microscopy of systemic antisense oligonucleotide (ASO) drug activity. TR;HSA$^{LR}$ bi-transgenic mice treated with saline or ASO 445236, which is designed to induce cleavage and degradation of pathogenic RNA containing expanded CUG repeats[4] (N = 2 each group). ASO administration was by subcutaneous injection of 25 mg/kg twice weekly for four weeks (eight doses), then once every two weeks for two weeks, for a total of 10 doses (see Fig. 5). Shown are representative images of quantitative DsRed and GFP fluorescence in gastrocnemius muscles under general anesthesia on Day 0 (pre-treatment) and Day 42. Fluorescence intensity range = 0 - 4095 grayscale units. Bars = 2 millimeters.

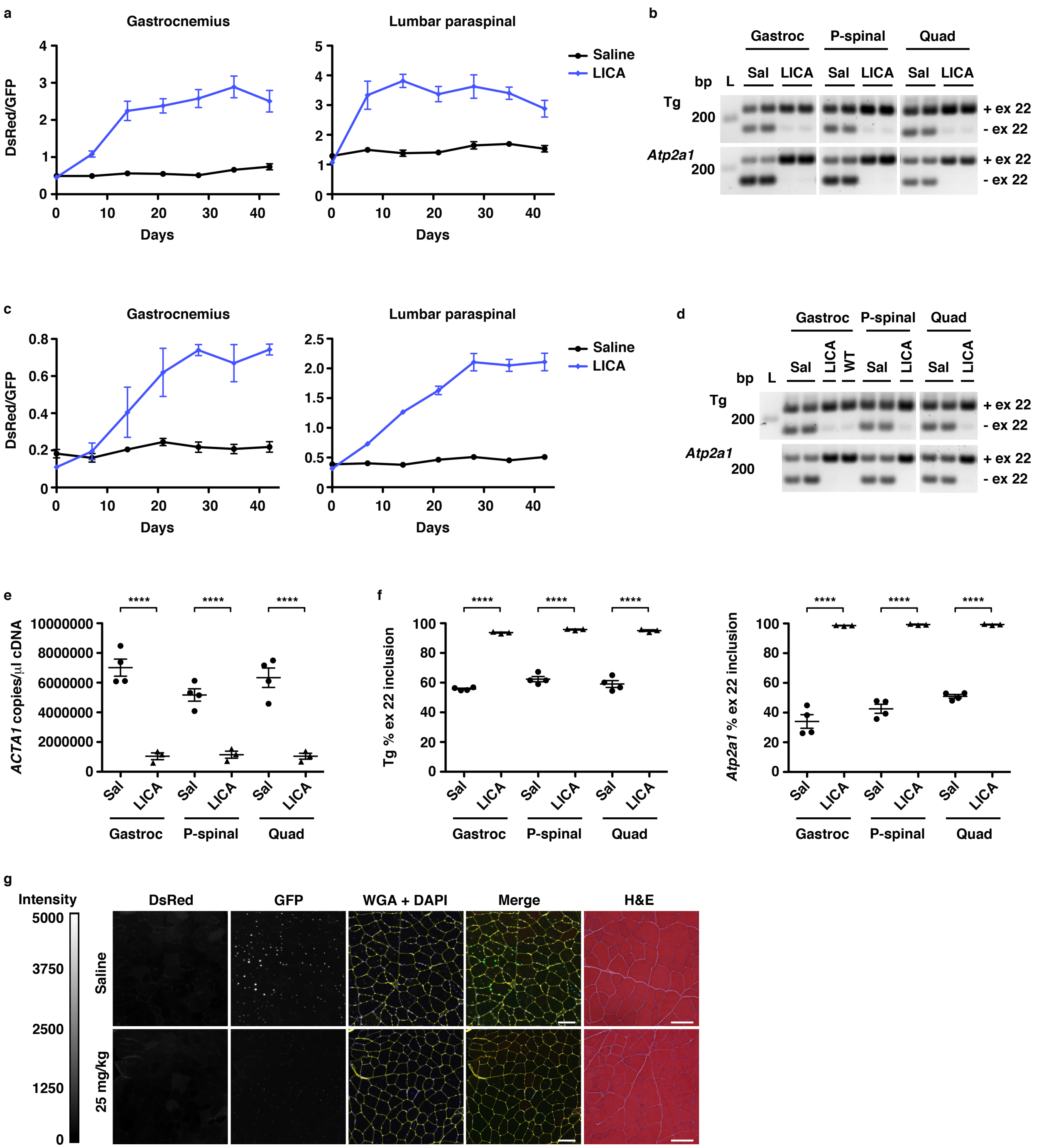


**Supplementary Figure 7** *In vivo* activity of a novel ligand-conjugated antisense (LICA) oligonucleotide. We screened *in vivo* activity of a LICA oligonucleotide targeting *ACTA1* by subcutaneous injection of TR;HSA$^{LR}$ mice with saline (Sal; N = 4 total) or 25 mg/kg (LICA; N = 3 total) twice per week for four weeks (eight total doses) and monitored DsRED/GFP ratios by *in vivo* fluorescence microscopy or spectroscopy for six weeks. (**a**) Serial *in vivo* fluorescence microscopy measurements in gastrocnemius (left) and lumbar paraspinal muscles (right) in mice treated with saline (black circles) or LICA oligo (blue triangles) (N = 2 each). Error bars indicate mean ± s.e.m. (**b**) RT-PCR analysis of exon 22 alternative splicing of the transgene (Tg) and

mouse *Atp2a1* in gastrocnemius, lumbar paraspinal, and quadriceps muscles collected on imaging Day 42. L = DNA ladder; bp = base pairs. (**c**) Serial *in vivo* fluorescence spectroscopy measurements in gastrocnemius (left) and lumbar paraspinal muscles (right) in mice treated with saline (N = 2) or LICA oligo (N = 1). Error bars indicate mean ± s.e.m. (**d**) RT-PCR analysis of exon 22 alternative splicing of the transgene (Tg) and mouse *Atp2a1* in gastrocnemius, lumbar paraspinal, and quadriceps muscles collected on imaging Day 42. FVB WT gastrocnemius served as a control. L = DNA ladder; bp = base pairs. (**e**) ddPCR analysis of *ACTA1* expression in muscles combined from (**b**) and (**d**). **** $P < 0.0001$; two-way ANOVA. (**f**) Quantitation of combined splicing results shown in (**b**) and (**d**). **** $P < 0.0001$; two-way ANOVA. (**g**) Representative images of quantitative DsRED and GFP fluorescence in muscle cryosections from saline or LICA-treated mice. Muscle fiber membranes are highlighted with Alexa 647-wheat germ agglutinin (WGA; pseudocolored yellow) and nuclei are labeled with DAPI (blue). Merge = DsRed (red) + GFP (green) + WGA + DAPI. Fluorescence images are the extended focus of deconvolved Z-series. Fluorescence intensity range = 0 - 5000 grayscale units. Bars = 100 μm.

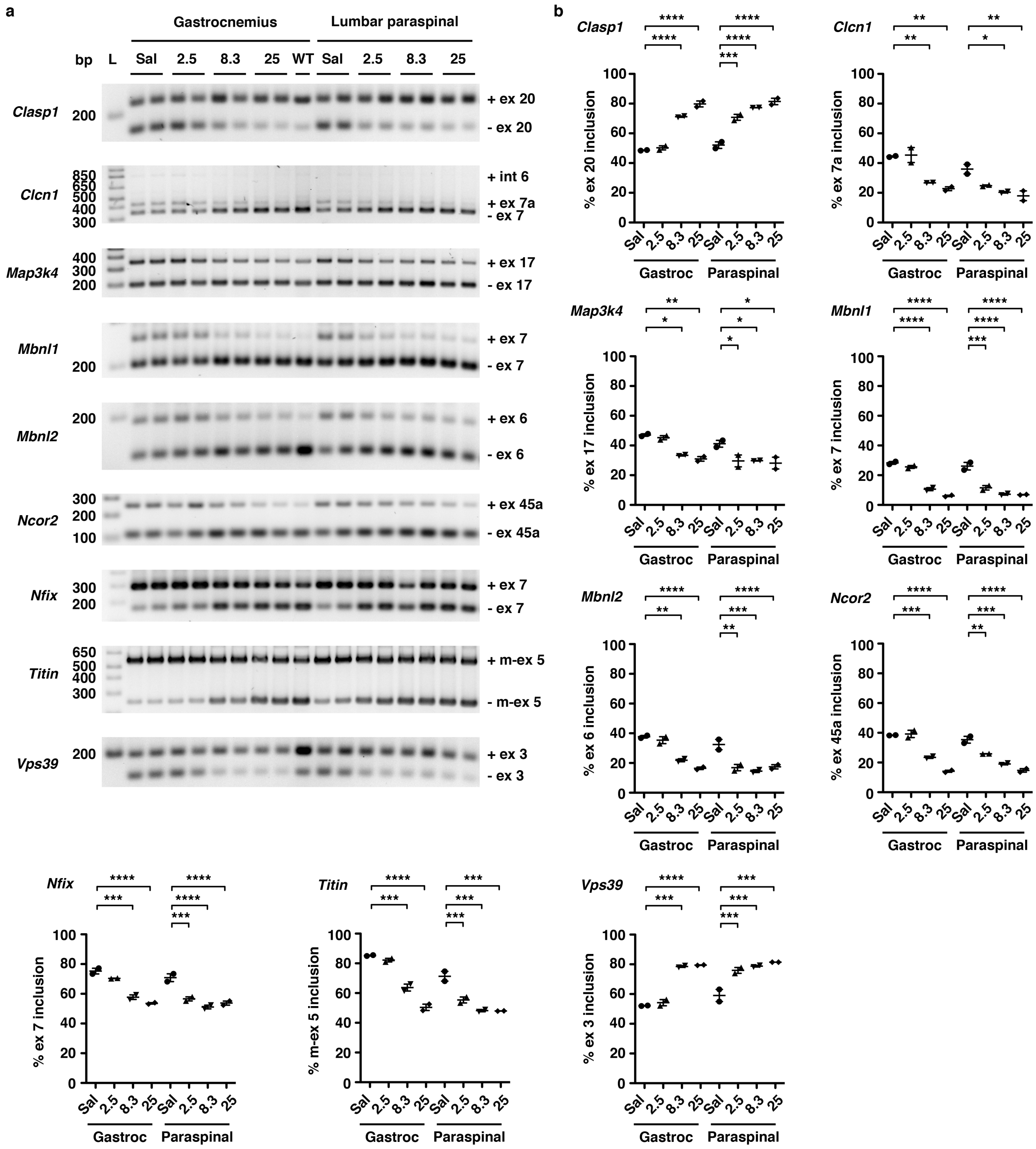


**Supplementary Figure 8** Dose-dependent activity of a novel ligand-conjugated antisense (LICA) oligonucleotide. We treated TR;HSA$^{LR}$ mice with subcutaneous injection of saline (sal) or *ACTA1*-targeting LICA oligo 992948 at doses of either 2.5 mg/kg, 8.3 mg/kg, or 25 mg/kg twice weekly for four weeks (N = 2 each group) and monitored DsRed and GFP fluorescence by non-invasive fluorescence spectroscopy (see Fig. 6). After the final spectroscopy data acquisition on Day 42 of treatment, we analyzed alternative splicing in gastrocnemius and lumbar paraspinal muscles by RT-PCR. Muscles from an FVB wild-type (WT) served as untreated controls. (**a**) Gel electrophoresis images of splicing patterns of the transcripts *Clasp1* exon 20,

*Clcn1* exon 7a, *Map3k4* exon 17, *Mbnl1* exon 7, *Mbnl2* exon 6, *Ncor2* exon 45a, *Nfix* exon 7, *Titin* m-line exon 5, and *Vps39* exon 3 in saline and LICA oligo-treated muscles. L = DNA ladder; bp = base pairs. (**b**) Quantitation of the results in (**a**). Error bars indicate mean ± s.e.m. **** $P < 0.0001$, *** $P < 0.001$, ** $P < 0.01$, * $P < 0.05$; two-way ANOVA.

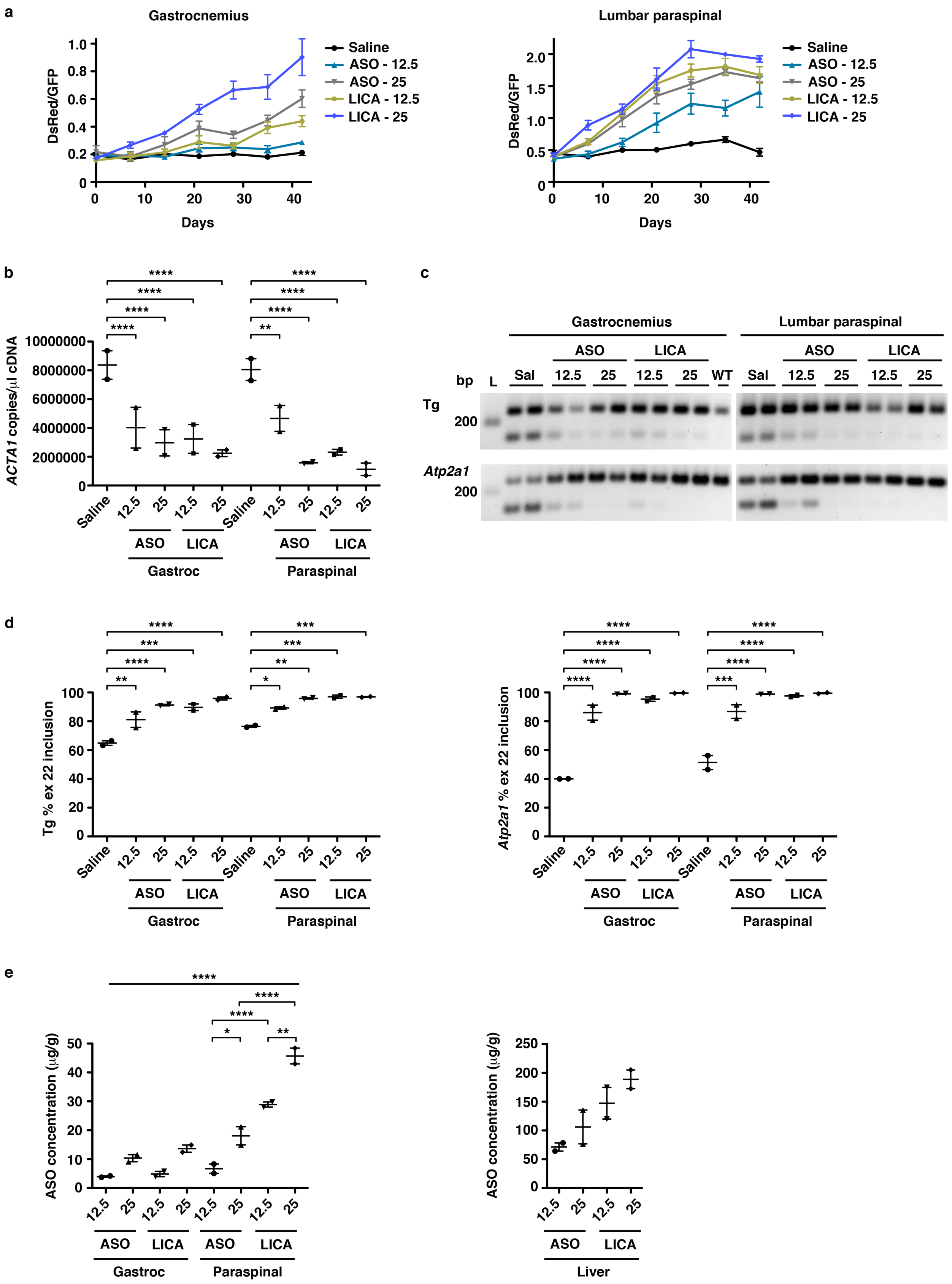
a
Gastrocnemius
DsRed/GFP
Days
Saline
ASO - 12.5
ASO - 25
LICA - 12.5
LICA - 25
Lumbar paraspinal
b
ACTA1 copies/μl cDNA
Saline
12.5
25
ASO
LICA
Gastroc
Paraspinal
c
Gastrocnemius
Lumbar paraspinal
bp
L
Sal
WT
Tg
Atp2a1
200
d
Tg % ex 22 inclusion
Atp2a1 % ex 22 inclusion
e
ASO concentration (μg/g)
Liver

**Supplementary Figure 9** LICA oligonucleotide modifications enhance *in vivo* target engagement over the unconjugated parent ASO. We treated TR;*HSA*$^{LR}$ mice with saline, LICA oligo 992948 (LICA), or ASO 445236 (ASO), which is the unconjugated parent of the LICA oligo 992948 that targets the identical *ACTA1* sequence[4]. ASOs were administered by subcutaneous injection of 12.5 or 25 mg/kg twice weekly for four weeks (N = 2 each group) (eight total doses). (**a**) DsRed/GFP quantitative fluorescence in gastrocnemius (left) and lumbar paraspinal muscles (right) by serial *in vivo* spectroscopy of mice treated with saline (black circles), ASO 12.5 mg/kg dose (teal triangles), ASO 25 mg/kg dose (gray triangles), LICA 12.5 mg/kg dose (yellow pentagons), or LICA 25 mg/kg dose (blue diamonds). Error bars indicate mean ± s.e.m. (**b**) After spectroscopy measurements on Day 42, muscles were dissected and *ACTA1*-CUG$^{exp}$ transcripts quantitated (copies per microliter cDNA) by droplet digital PCR (ddPCR) in gastrocnemius (Gastroc) and lumbar paraspinal muscles. Error bars indicate mean ± s.e.m. **** $P < 0.0001$, ** $P < 0.01$; two-way ANOVA. (**c**) RT-PCR analysis of exon 22 alternative splicing of the transgene (Tg) and mouse *Atp2a1* in gastrocnemius, lumbar paraspinal, and quadriceps muscles collected on imaging Day 42. FVB WT gastrocnemius served as a control. L = DNA ladder; bp = base pairs. (**d**) Quantitation of the results shown in (**c**). Error bars indicate mean ± s.e.m. **** $P < 0.0001$, *** $P < 0.001$, ** $P < 0.01$, * $P < 0.05$; two-way ANOVA. (**e**) ASO drug concentration (micrograms/gram) at Day 42 in gastrocnemius (Gastroc) and lumbar paraspinal muscles (left) and liver (right) as measured by high pressure liquid chromatography coupled with tandem mass spectrometry. Error bars indicate mean ± s.e.m. **** $P < 0.0001$ (overall effect of treatment, gastroc vs. paraspinal), (paraspinal, ASO - 12.5 vs. ASO - 25), and (paraspinal, LICA - 12.5 vs. LICA - 25); ** $P < 0.01$ (paraspinal, LICA - 12.5 vs. LICA - 25); * $P < 0.05$ (paraspinal, ASO - 12.5 vs. ASO - 25); two-way ANOVA.

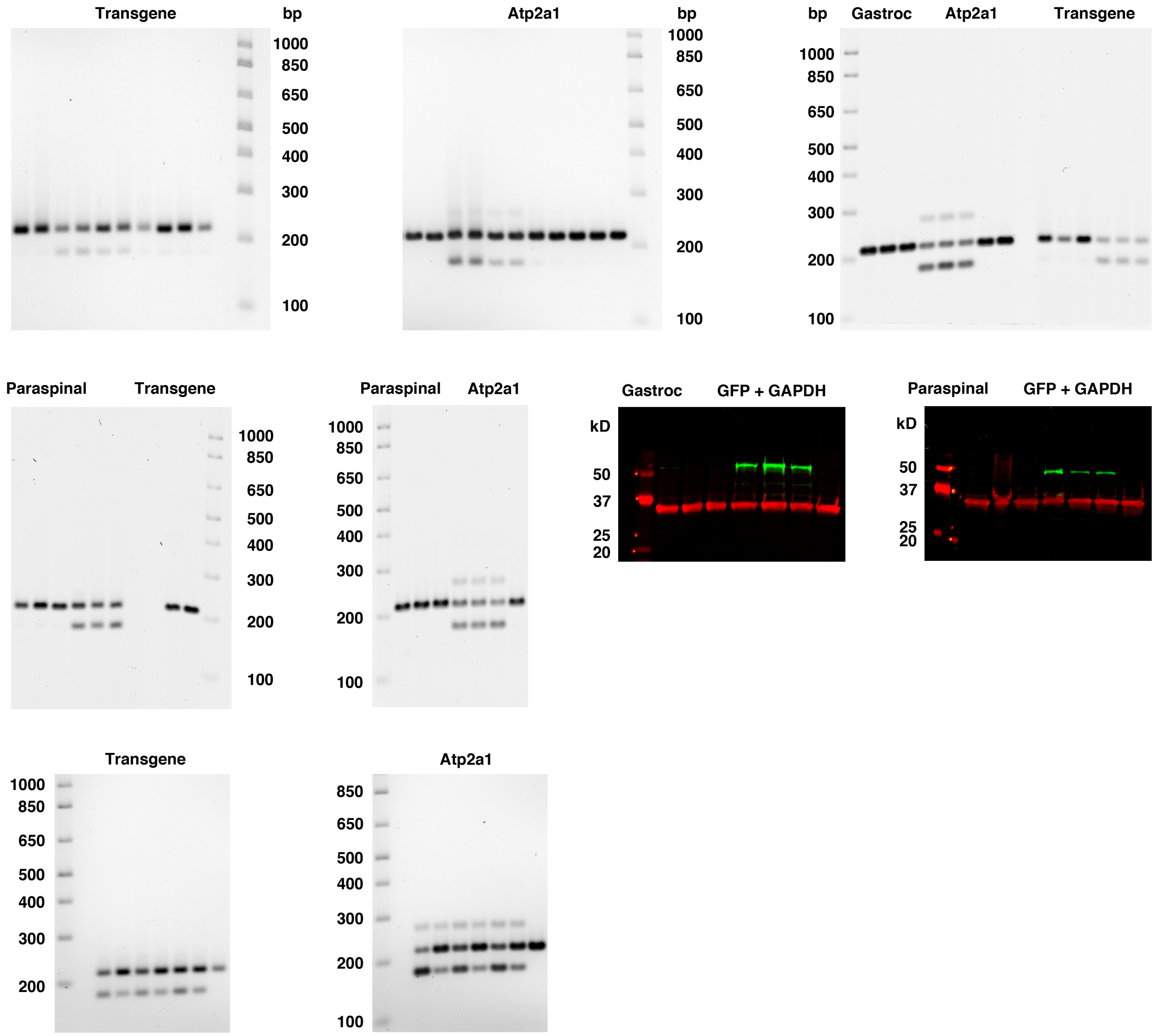


**Supplementary Figure 10** Uncropped gel images of alternative splicing outcomes shown in Figures 1 - 3, and uncropped Western blot images of GFP and GAPDH protein expression shown in Figure 2. The DNA molecular weight marker is the 1 Kb Plus DNA Ladder (Invitrogen). The protein molecular weight marker is the Kaleidoscope Precision Plus Protein Standard (Bio-Rad). bp = base pairs. kD = kilodaltons.

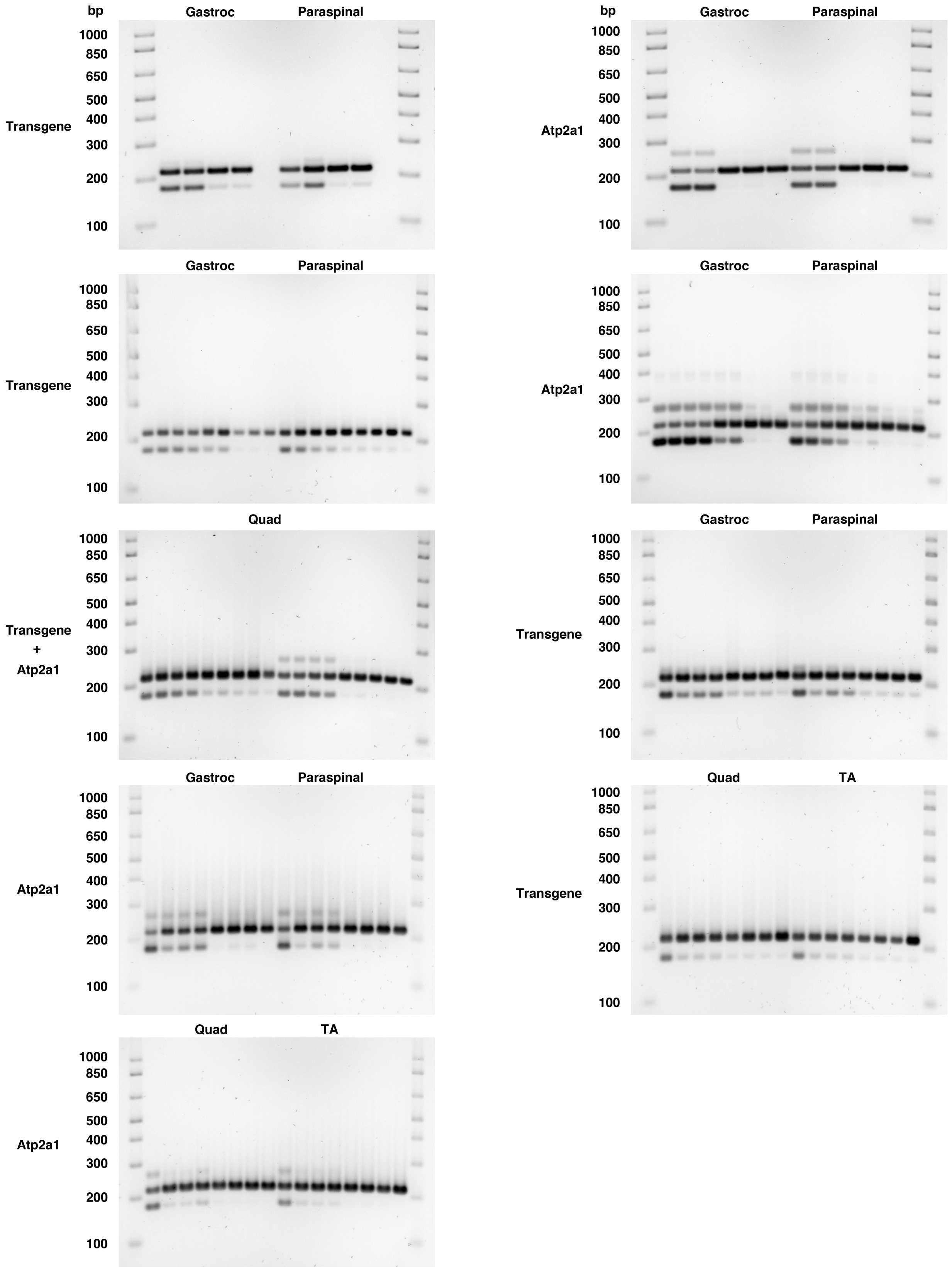


**Supplementary Figure 11** Uncropped gel images of alternative splicing outcomes shown in Figures 5 - 7. The molecular weight marker is the 1 Kb Plus DNA Ladder (Invitrogen). bp = base pairs.

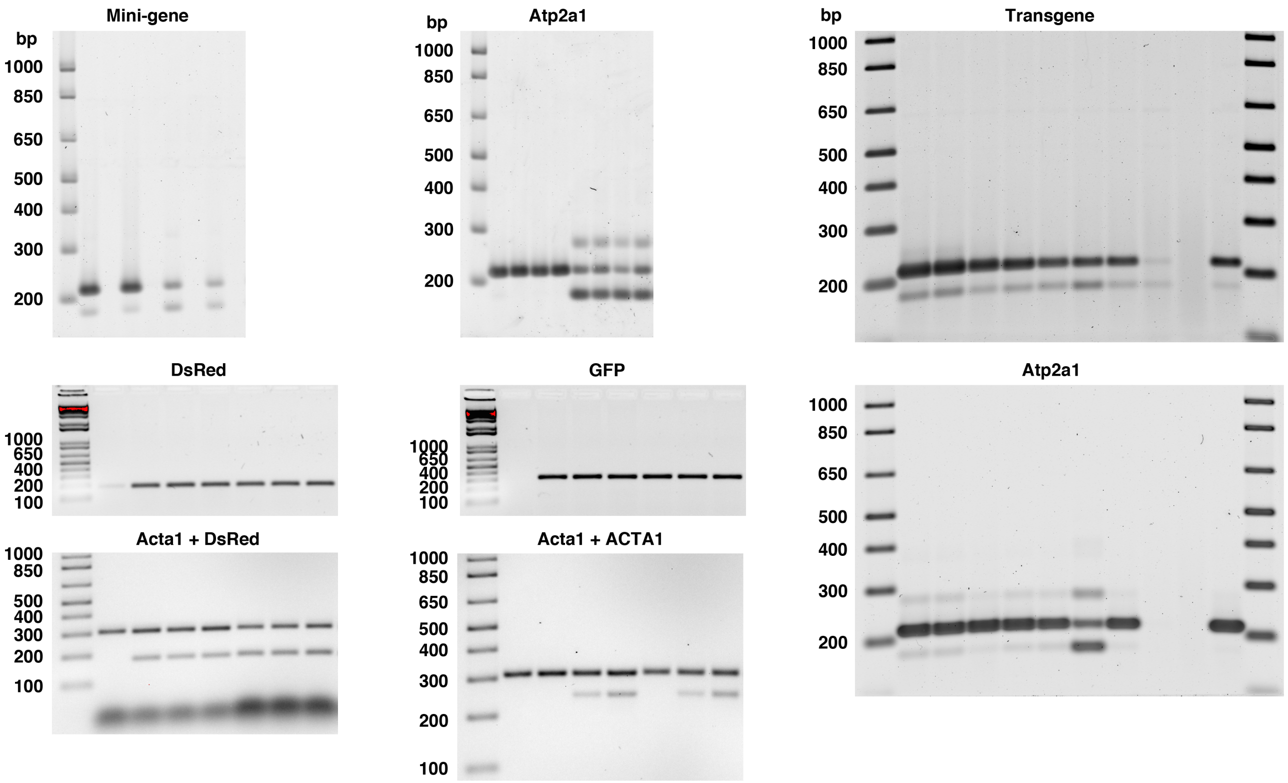


**Supplementary Figure 12** Uncropped gel images of alternative splicing outcomes shown in Supplementary Figures 2 and 3, and genotyping outcomes shown in Supplementary Figure 3. The molecular weight marker is the 1 Kb Plus DNA Ladder (Invitrogen). bp = base pairs.

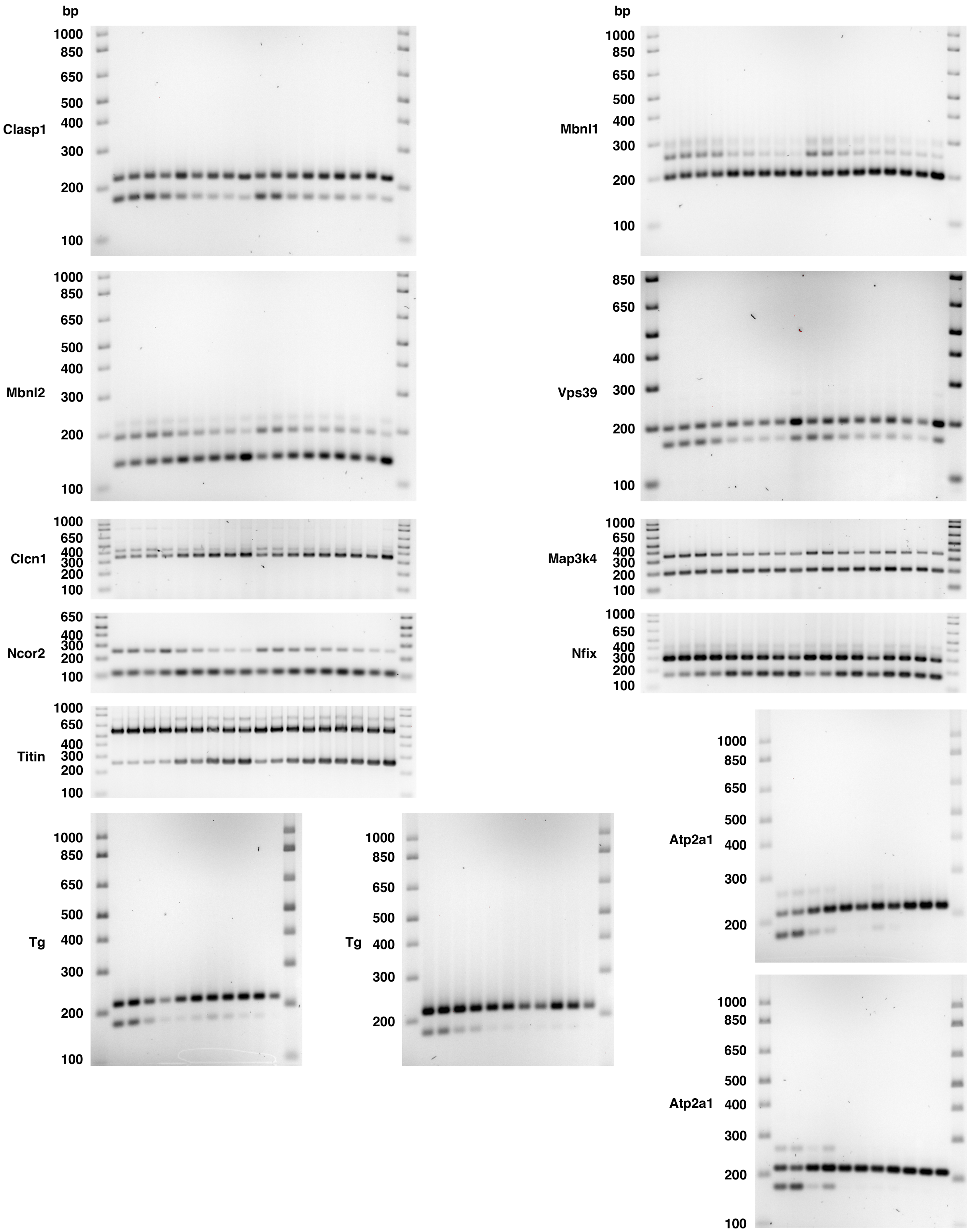


**Supplementary Figure 13** Uncropped gel images of alternative splicing outcomes shown in Supplementary Figures 8 and 9. The molecular weight marker is the 1 Kb Plus DNA Ladder (Invitrogen). Tg = transgene. bp = base pairs.

| Mouse ID | TR transgene | | | $HSA^{LR}$ transgene | | | Overall genotype |
|---|---|---|---|---|---|---|---|
| | *Acta1* (%) | DsRed (%) | TR zygosity | *Acta1* (%) | *ACTA1* (%) | $HSA^{LR}$ zygosity | |
| 1 | 100 | 0 | -/- | 100 | 0 | -/- | WT |
| 2 | 72 | 28 | +/- | 100 | 0 | -/- | TR hemi;WT |
| 3 | 73 | 27 | +/- | 86 | 14 | +/- | TR hemi;$HSA^{LR}$ hemi |
| 4 | 72 | 28 | +/- | 76 | 24 | +/+ | TR hemi;$HSA^{LR}$ hom |
| 5 | 58 | 42 | +/+ | 100 | 0 | -/- | TR hom;WT |
| 6 | 58 | 42 | +/+ | 86 | 14 | +/- | TR hom;$HSA^{LR}$ hemi |
| 7 | 56 | 44 | +/+ | 72 | 28 | +/+ | TR hom;$HSA^{LR}$ hom |

**Supplementary Table 1** Band densitometry results and overall genotype for all seven mice shown in Supplementary Fig. 2. Hemi = hemizygous; Hom = homozygous.

| | Channel | ROI | ROI Location | ROI Area (µm²) | Min | Max | Mean | Exposure time (sec) | Mean/exposure time | Mean - background | DsRed/ GFP |
|---|---|---|---|---|---|---|---|---|---|---|---|
| Day 7 | DsRed | Blue | Left gastroc | 3973380.94 | 1337 | 2727 | 1955.74 | 5 | 391.15 | 265.93 | 3.30 |
| | DsRed | Green | Right gastroc | 3973380.94 | 702 | 1515 | 1039.43 | 5 | 207.89 | 82.66 | 0.51 |
| | DsRed | Red | Background | 1463723.42 | 572 | 668 | 626.11 | 5 | 125.22 | 0 | |
| | GFP | Blue | Left gastroc | 3973380.94 | 1064 | 1992 | 1263.08 | 5 | 252.62 | 80.50 | |
| | GFP | Green | Right gastroc | 3973380.94 | 1109 | 2379 | 1673.66 | 5 | 334.73 | 162.62 | |
| | GFP | Red | Background | 1463723.42 | 795 | 908 | 860.57 | 5 | 172.11 | 0 | |

| | Channel | ROI | ROI Location | ROI Area (µm²) | Min | Max | Mean | Exposure time (sec) | Mean/exposure time | Mean - background | DsRed/ GFP |
|---|---|---|---|---|---|---|---|---|---|---|---|
| Day 21 | DsRed | Blue | Left gastroc | 3973380.94 | 1467 | 2515 | 2027.61 | 6 | 337.94 | 223.90 | 3.76 |
| | DsRed | Green | Right gastroc | 3973380.94 | 1656 | 3085 | 2501.3 | 6 | 416.88 | 302.85 | 3.39 |
| | DsRed | Red | Background | 1426716.44 | 634 | 737 | 684.23 | 6 | 114.04 | 0 | |
| | GFP | Blue | Left gastroc | 3973380.94 | 941 | 1442 | 1089.82 | 5 | 217.96 | 59.49 | |
| | GFP | Green | Right gastroc | 3973380.94 | 1023 | 2926 | 1238.88 | 5 | 247.78 | 89.30 | |
| | GFP | Red | Background | 1426716.44 | 709 | 902 | 792.38 | 5 | 158.48 | 0 | |

**Supplementary Table 2** Quantitation of *in vivo* fluorescence by microscopy. Using *in vivo* fluorescence microscopy and Volocity software (Perkin Elmer; see Methods), we quantitated fluorescence within regions of interest (ROIs) in TR transgenic mice on Days 7 and 21 after acute muscle injury with 1.2% barium chloride. The position of each ROI is shown in Supplementary Fig. 3. For each muscle, ROI location was identical for the DsRed and GFP channels. To calculate DsRed/GFP fluorescence ratios, the mean fluorescence was divided by the image exposure time in seconds, followed by subtraction of the background fluorescence and division of the corrected DsRed fluorescence by the corrected GFP fluorescence.

| **COMPONENT** | **PART NUMBER/VENDOR/DESCRIPTION** |
|---|---|
| Spectroscopy Fibers | Thorlabs M28L01 (400 µm core, 0.39 NA) |
| Broadband White lamp | Ocean Optics HL-2000 |
| Stepper-Motor-Driven Filter Wheels | Thorlabs FW102C |
| Spectrometer | Ocean Optics QE65PRO-FL |
| **Diode Lasers (50 mW output)**<br>488 nm laser<br>532 nm laser | <br>Coherent OBIS 488 nm LX 50 mW<br>Oxxius S.A. LMX-532S-50-COL-PP |
| Power Detector + Meter | Thorlabs Photodiode power sensor + detector (PM200) |
| Miscellaneous Optical Accessories | Thorlabs |
| 3 x 1 Fiber Switch | Piezosystem Jena, Inc. F-143-10IR |
| **Laser Line Filters**<br>488 nm MaxLine laser clean-up filter<br>532 nm MaxLine laser clean-up filter | <br>Semrock LL01-488-12.5<br>Semrock LL01-532-12.5 |
| **Long Pass Filters**<br>488 nm RazorEdge long-pass filter<br>532 nm EdgeBasic long-pass filter | **Diameter: 25 mm (matches filter wheel slot)**<br>Semrock LP02-488RU-25<br>Semrock BLP01-532R-25 |
| 2 x Fiber Coupling Accessories | OZ Optics Free Space Laser Fiber Coupler |
| Data Acquisition Board | National Instruments USB-6008 |
| Labview Software License | National Instruments |
| Laptop | Apple Macbook Pro |

**Supplementary Table 3** List of components for the *in vivo* spectroscopy system.

| **Gene** | **Left primer (5′ - 3′)** | **Right primer (5′ - 3′)** | **Target exon(s)** | **+ ex size (nt)** | **- ex size (nt)** |
|---|---|---|---|---|---|
| Tg-*ATP2A1* | CCGAGCCGAGAGTAGCAGTTG | GCACACCGGTGGTGTTGTTG | 22 | 240 | 199 |
| *Atp2a1* | GCTCATGGTCCTCAAGATCTCAC | GGGTCAGTGCCTCAGCTTTG | 22 | 218 | 176 |
| *Clasp1* | GTCGACGACAGGATCTCTCC | GAGCTCTGCCGTCTCGTG | 20 | 198 | 174 |
| *Clcn1* | TGAAGGAATACCTCACACTCAAGG | CACGGAACACAAAGGCACTG | 7a | 424 | 345 |
| *Map3k4* | CAACAGAATCAGCGATGCCATC | TGGTCTGGCTGATGAGTGTTCG | 17 | 355 | 199 |
| *Mbnl1* | ACCTGCAAGCCAAGATCAAG | TGTTGGCTAGAGCCTGTTGG | 7 | 255 | 201 |
| *Mbnl2* | TCACCCTCCTGCACACTTG | TCTTTGGTAAGGGATGAAGAGC | 6 | 194 | 140 |
| *Ncor2* | CAGGCGGTGCAAGAACAC | TTCGGCTGCTAGGTCTGC | 45a | 253 | 112 |
| *Nfix* | TCGACGACAGTGAGATGGAG | CTGGATGATGGACGTGGAAG | 7 | 238 | 115 |
| *Titin* | GTGTGAGTCGCTCCAGAAACG | CCACCACAGGACCATGTTATTTC | 5 | 556 | 253 |
| *Vps39* | AATGGCTTCTTGTTGGAACC | GCTGACCAGAATCTTAAACTGG | 3 | 197 | 164 |

**Supplementary Table 4** PCR primers that we used to measure alternative splicing. Tg-*ATP2A1* = transgene human *ATP2A1*. We designed primers for Tg-*ATP2A1*, *Mbnl2*, and *Vps39* using Primer3 software[5, 6]. Primers for *Clcn1*, *Titin*, *Clasp1*, *Map3k4*, *Mbnl1*, *Ncor2*, *Nfix*, and endogenous mouse *Atp2a1* were published previously[7-9].

**agatctatcgat**aagctttctgtaaggaaaggtaagagttgaactgagcaagagttttgaaaaatagtgacaatcccattctcctttggaatgcgcacaaatattgaggtatcc
agtgaacggcagcaaatttcctaccttcaaggcccaaatgtaagctagtccccttacgttacatgcagctcatttgctaagtggtttttttctagtatctccactactcgctga
cacaggaggacacaggatgttaaaaaggaaatacagttctgtcaattattcacttactctccaaaatacttggaagaactaaatatggaaccataggagactttatcctcaccg
catagtccctatactagtcaaactccttattttttaattgatcatttttaggaaggtagcattttattcactagaacatttttgttaatacttgtttattttttgggatgaactg
ccatgatgtgggctacagaggagggtcgcatatgcttccatccccctttagagaatccacacctgtcccagttgctgggttccactaccaaaagtgaattgcaactattttag
gagcacttaagcacatccgaaaaatgagtgattctgttctggcccacaccacatcactgatgtaccccttaaagcatgtccctgagttcatcacagaagactgctcctcctgt
gccctccacaaggttagaactgtccttgtcttagggaaaaaggagagagagagagagagagagagagagagagagagagagagagagagagagagagggacaggcaccaactgg
gtaacctctgctgacccccactctactttaccataagtagctccaaatccttctagaaaatctgaaaggcatagccccatatatcagtgatataaatagaacctgcagcaggct
ctggtaaatgatgactacaaggtggactgggaggcagcccggccttggcaggcatcatcctctaaatataaagatgagtttgttcagcctttgcagaaggaaaaactgccaccc
atcctagagtgccgcgtccttgtcccccacccccctccaatttattgggaggaaggaccagctaagcctcatctaggaagagcccctcacccatctccacctccactccaggtc
tagccagtcctgggttgtgacccttgtctttcagccccaggagagggacacacatagtgccaccaaagaggctgggggagggcctcagcccaccaaaacctggggccagtgcgt
cctacaggaggggaaccctcaccccttcaatccctttaggagacccaagggcgctgcgcgtccctgaggcggacagctccgtgtgctcaggctttgcgcctgacaggcctatcc
ccgggagcccccgcgcctcctccccggcgctccgccctcgcctcccccgccagttgtctatcctgcgacagctgcgcgccctccggccgccggtggccctctgtgcggtgggg
gaaggggtcgacgtggctcagctttttggattcagggagctcggggggtgggaagagagaaatggagttccaggggcgtaaaggagagggagttcgccttccttcccttcctgag
actcaggagtgactgcttctccaatcctcccaagcccaccactccacacgactccctcttcccggtagtcgcaagtgggagtttggggatctgagcaaagaacccgaagaggag
ttgaaatattggaagtcagcagtcaggcaccttcccgagcgcccagggcgctcagagtggacatggttggggaggcctttgggacaggtgcggttcccggagcgcaggcgcaca
catgcacccaccggcgaacgcggtgaccctcgccccaccccatcccctccggcgggcaactgggtcgggtcaggaggggcaaacccgctagggagacactccatatacggcccg
gcccgcgttacctgggaccgggccaacccgctccttctttggtcaacgcaggggacccgggcgggggcccaggccgcgaaccggccgagggaggggggctctagtgcccaacacc
caaatatggctcgagaagggcagcgacattcctgcggggtggcgcggagggaatgcccgcgggctatataaaacctgagcagagggacaagcggCCACCGCAGCGGACAGCGCC
AAGTGAAGCCTCGCTTCCCCTCCGCGGCGACCAGGGCCCGAGCCGAGAGTAGCAGTTGTAGCTACCCGCCCAGgtagggcaggagttgggaggggacagggggacagggcacta
ccgaggggaacctgaaggactccggggcagaacccagtcggttcacctggtcagccccaggcctgcgccctgagcgctgtgcctcgtctccggagccacacgcgctttcaagct
ttttggcgtgtaatacaaaatgacattcctaggctcacctcacaggagcttgaaagaagaacccaaagatatttaagggacagtttggcaaggtttttagaggaaactacttgg
acagtaattaatgctcctgttaattggtataactctttacaagattactactctactttgtctcccattaggcctacaatggtgagacaagtagccaacagggaagggttgcaa
atatcatttgggcacacctatgataatattgatgaagcagacagtattcagcaagtaactgagaggtgggaagctcaaagccaaagtcctaatgtgcagtcaggtgaatttatt
gaaaaatttgaggctcctggtggtgcaaatcaaagaactgctcctcagtggatgttgcctttacttctaggcctgtacggaagtgttacttctgctctaaaccggatcgatccc
tcgagtctaga**gcggccgccaccATGG**ATTACAAGGATGACGATGACAAGtctaga**ggatcc**a**AGAGG**gtaaggagtgccctctctgtcccaagccctggccccaccacagccc
cttccccatgacgccgcccccgccccgccccgtactttgcaggtggtaagtttctcagccctggcaggacctgtgtccgccccgttccccctgcgcctgcaggggccacatctc
cggggcagccccactgcctcctcagcccccacagcccctatagcccccatgccacctccctgccttgataacagtgcctcttgtcctctctggccatag**GATAACTGTTCCCCTCCTCCATCTCTGAGCCCGTGTCACAG**gtatcaccccttcttgccctcagcccagctgctgtgcccctgccacccgcgcccccctcagccccttgcgcgtcgcatccaaggtcac
ttgtgctcgcagctccacctggagccgttgccactgctgctgctgcgcttccagtcagggtgggccactggcctcccactgggcgtcagtttggctcccaggccctgggcagtg
ccagcctctgggcccgtctgctgcgctgcgttgcgctggctgtgtgctgggctgtctttgctgtggggctgcagtggggggggggcggggtgtctggggacgcaggtgagtagg
ggagaaactggcagggtggtaagcttctgagcctccaggtaagtgcgtgcctgggcgatgcacctgggagggacctcgctgccctcctgccgcctgcctcatcccttctctttc
cccttccag**ATggatcc**CCAACAACACC**accggt**GTGcgctcctccaagaacgtcatcaaggagttcatgcgcttcaaggtgcgcatggagggcaccgtgaacggccacgagtt
cgagatcgagggcgagggcgagggccgcccctacgagggccacaacaccgtgaagctgaaggtgaccaagggcggcccccctgcccttcgcctgggacatcctgtccccccagtt
ccagtacggctccaaggtgtacgtgaagcaccccgccgacatccccgactacaagaagctgtccttccccgagggcttcaagtgggagcgcgtgatgaacttcgaggacggcgg
cgtggtgaccgtgacccaagactcctccctgcaggacggctgcttcatctacaaggtgaagttcatcggcgtgaacttcccctccgacggccccgtaatgcagaagaagaccat
gggctgggaggcctccaccgagcgcctgtaccccgcgacggcgtgctgaagggcgagatccacaaggccctgaagctgaaggacggcggccactacctggtggagttcaagtc
catctacatggccaagaagcccgtgcagctgcccggctactactacgtggactccaagctggacatcacctcccacaacgaggactacaccatcgtggagcagtacgagcgcac
cgagggccgccaccacctgttcctgTAGa**ccgcgg**tGTGagcaagggcgaggagctgttcaccggggtggtgcccatcctggtcgagctggacggcgacgtaaacggccacaag
ttcagcgtgtccggcgagggcgagggcgatgccacctacggcaagctgaccctgaagttcatctgcaccaccggcaagctgcccgtgccctggcccaccctcgtgaccaccctg
acctacggcgtgcagtgcttcagccgctaccccgaccacatgaagcagcacgacttcttcaagtccgccatgcccgaaggctacgtccaggagcgcaccatcttcttcaaggac
gacggcaactacaagacccgcgccgaggtgaagttcgagggcgacaccctggtgaaccgcatcgagctgaagggcatcgacttcaaggaggacggcaacatcctggggcacaag
ctggagtacaactacaacagccacaacgtctatatcatggccgacaagcagaagaacggcatcaaggtgaacttcaagatccgccacaacatcgaggacggcagcgtgcagctc
gccgaccactaccagcagaacacccccatcggcgacggccccgtgctgctgcccgacaaccactacctgagcacccagtccgccctgagcaaagaccccaacgagaagcgcgat
cacatggtcctgctggagttcgtgaccgccgccgggatcactctcggcatggacgagctgtacaagtaa**gggccc**gtttaaacccgctgatcagcctcgactgtgccttctagt
tgccagccatctgttgtttgcccctcccccgtgccttccttgaccctggaaggtgccactcccactgtcctttcc**taataaaa**tgaggaaattgcatcgcattgtctgagtagg
tgtcattctattctggggggtggggtggggcaggacagcaagggggaggattgggaagacaatagcag**gcatgc**

**agatct** : *Bgl* II restriction site (5′ end of transgene fragment)

**atcgat** : *Cla* I restriction site (5′ end of HSA promoter/enhancer/Vp1 splice acceptor[3])

**gcggccgc** : *Not* I restriction site (3′ end of HSA promoter/enhancer/Vp1 splice acceptor[3])

**ggatcc** : *Bam* HI restriction sites (5′ and 3′ ends of the *ATP2A1* minigene)

**GATAACTGTTCCCCTCCTCCATCTCTGAGCCCGTGTCACAG** : *ATP2A1* exon 22 (modified from 42 to 41 bp)

**accggt** : *Age* I restriction site (5′ end of DsRed[1])

**ccgcgg** : *Sac* II restriction site (3′ end of DsRed; 5′ end of GFP[1])

**gggccc** : *Apa* I restriction site (3′ end of GFP[1])

**gcatgc** : *Sph* I restriction site (3′ end of transgene fragment)

**Supplementary Note 1** TR sequence with HSA promoter/enhancer (between the *Cla* I - *Not* I sites) and the *ATP2A1* minigene (between the *Bam* HI sites) (see Fig. 1a). The *Bgl* II - *Sph* I fragment comprising the transgene = 5.316 kb.

## Supplementary References


1. Orengo, J.P., Bundman, D. & Cooper, T.A. A bichromatic fluorescent reporter for cell-based screens of alternative splicing. *Nucleic Acids Res* **34**, e148 (2006).
2. Bertoni, C. et al. Enhancement of plasmid-mediated gene therapy for muscular dystrophy by directed plasmid integration. *Proc Natl Acad Sci U S A* **103**, 419-424 (2006).
3. Crawford, G.E. et al. Assembly of the dystrophin-associated protein complex does not require the dystrophin COOH-terminal domain. *J Cell Biol* **150**, 1399-1410 (2000).
4. Wheeler, T.M. et al. Targeting nuclear RNA for in vivo correction of myotonic dystrophy. *Nature* **488**, 111-115 (2012).
5. Koressaar, T. & Remm, M. Enhancements and modifications of primer design program Primer3. *Bioinformatics* **23**, 1289-1291 (2007).
6. Untergasser, A. et al. Primer3--new capabilities and interfaces. *Nucleic Acids Res* **40**, e115 (2012).
7. Wheeler, T.M., Lueck, J.D., Swanson, M.S., Dirksen, R.T. & Thornton, C.A. Correction of ClC-1 splicing eliminates chloride channelopathy and myotonia in mouse models of myotonic dystrophy. *J Clin Invest* **117**, 3952-3957 (2007).
8. Lin, X. et al. Failure of MBNL1-dependent post-natal splicing transitions in myotonic dystrophy. *Hum Mol Genet* **15**, 2087-2097 (2006).
9. Antoury, L. et al. Analysis of extracellular mRNA in human urine reveals splice variant biomarkers of muscular dystrophies. *Nat Commun* **9**, 3906 (2018).
